# Longitudinal User Engagement with Microinteraction Ecological Momentary Assessment (μEMA)

**DOI:** 10.1145/3749541

**Published:** 2025-09-03

**Authors:** ADITYA PONNADA, SHIRLENE D WANG, JIXIN LI, WEI-LIN WANG, GENEVIEVE F DUNTON, DONALD HEDEKER, STEPHEN S INTILLE

**Affiliations:** Northeastern University, USA; Northwestern University, USA; Northeastern University, USA; University of Southern California, USA; University of Southern California, USA; University of Chicago, USA; Northeastern University, USA

**Keywords:** Ecological momentary assessment, smartwatch, microinteractions, longitudinal data collection

## Abstract

Microinteraction ecological momentary assessment (μEMA) is a type of EMA that uses single-question prompts on a smartwatch to collect real-world self-reports. Smaller-scale studies show that μEMA yields higher response rates than EMA for up to 4 weeks. In this paper, we evaluated μEMA’s longitudinal engagement in a 12-month study. Each participant completed EMA surveys (one smartphone prompt/hour for 96 days in 4-day bursts) and μEMA surveys (four smartwatch prompts/hour for the 270 days). Using data from 177 participants ( 1.37 million μEMA and 14.9K EMA surveys), we compared engagement across three groups: those who completed 12 months of EMA data collection(*Completed*), those who voluntarily withdrew after six months of EMA data collection (*Withdrew*), and those unenrolled by staff after six months of poor EMA response rates (*Unenrolled*). Compared to EMA, unenrolled participants were 2.25 times, those who withdrew were 1.65 times, and completed participants were 1.53 times more likely to answer μEMA prompts (*p* < 0.001). Regardless of response rates, μEMA was perceived as less burdensome than EMA (*p* < 0.001). These results suggest μEMA is a viable method for intensive longitudinal data collection, particularly for participants who find EMA unsustainable.

## 1 INTRODUCTION

Ecological momentary assessment (EMA), also known as the experience sampling method (ESM), has been used to gather real-time self-reported behaviors that sensors cannot yet measure (e.g., affect, motivations) [15, 73, 77] to build personalized behavior models [20, 59, 75] and novel user experiences [38]. In EMA, the user’s smartphone beeps or vibrates multiple times a day (at random [74] or triggered by sensors [19]). Each EMA interruption presents questions measuring behavior(s) of interest; typically, the prompt notifies the user about the availability of a survey consisting of several multiple-choice questions that can be answered on a smartphone. Compared to retrospective recall surveys, EMA has three benefits. It can be used to 1) ask about the current moment or the recent past to reduce recall bias, 2) capture temporal dynamics of behavior, and 3) collect data in natural settings, enabling better ecological validity [74].

Beyond observational studies, EMA also supports just-in-time adaptive interventions (JITAIs) [60] by providing self-report information to tailor the content [23], timing [49], or feedback on health behaviors [24], as well as to measure outcome constructs that require self-report (e.g., chronic pain) [29, 72]. As micro-randomized trials are increasingly used to optimize mobile health interventions, EMA could support user-in-the-loop personalization via active self-reporting [45]. EMA’s use has expanded from health behavior research (e.g., studying stress [81] and fatigue [76]) to human-computer interaction (HCI) [86], having been used to study smartphone use [88, 90], battery use in context [30], mobile information needs [11], and in-the-wild usability testing [13, 14]. In fact, large-scale intensive longitudinal data collection studies are becoming more mainstream in public health and human-computer interaction (e.g., [55, 91]), where self-report data are collected via smartphones for several months to years to build personalized behavior models.

The primary limitation of EMA is user burden. With each EMA prompt, participants must stop their activity, access their smartphone, and unlock the phone to answer multiple questions, causing burden [86]. These questions are often adapted from traditional paper-based surveys and are not necessarily optimized for mobile interactions in the wild [18]. As a result, EMA poses both a response burden (via cognitively complex questions [7]) and an interruption burden (through mobile notifications). Given the increasing interest in longitudinal measurement studies, a new challenge is adapting EMA to minimize burden and maximize information while measuring constructs at high frequency [8].

Microinteraction-based EMA, or micro-EMA (μEMA), addresses this density vs. user burden trade-off [34]. μEMA is a type of EMA that presents only one single question with single-tap answers with each prompt [34]. On a smartwatch, answering a μEMA question requires only a quick microinteraction — a 3–4 second glance-and-tap that can be completed amid other activities [3]. To achieve this microinteraction, the μEMA surveys are *intentionally* single questions, not question sets, designed to be cognitively simple to answer. In other words, EMA is a data collection paradigm that interrupts less, but asks for more information with each interruption. Whereas, μEMA interrupts more, but asks for small amounts of information with each interruption.

In prior work, using between-subject studies, μEMA has been shown to yield higher response rates and lower perceived burden than EMA (even with six times more prompting/hour), suggesting that μEMA may be a viable method for lightweight, *in-situ*, high-temporal-density self-reporting [34, 65]. **But how sustainable is μEMA in intensive longitudinal studies that last several months or more?** We present the first large-scale longitudinal evaluation of μEMA’s user engagement for high-frequency self-reporting across several months or more when data are collected in real-world settings. We measured user engagement in two parts — response rates and perceived burden. These two outcome variables capture the trade-off between μEMA (i.e., interrupt more, ask less) and EMA (i.e., interrupt less, ask more) methodologies, and address the following research questions:

- **RQ1:** How do μEMA response rates compare with EMA in intensive longitudinal data collection studies?
- **RQ2:** How burdensome do participants find answering questions using μEMA compared to EMA?

Our work extends the prior studies using μEMA in three ways. First, we compared μEMA and EMA on a larger scale with 177 participants (recruited nationally) each collecting data for up to *one year*, as compared to 33 participants collecting data for up to four weeks [33]. Second, we compared user engagement among participants exposed to both μEMA and EMA during data collection in a within-subject experiment, as compared to a between-subject experiment in prior work [33, 65]. The within-subject design permits estimating individuals’ relative preferences between EMA and μEMA. Third, we explored how μEMA engagement differs compared with participants’ engagement levels with EMA. We compare μEMA engagement among those participants who do not find EMA sustainable in a longitudinal study.

## 2 RELATED WORK

We build upon prior work on user engagement with EMA, EMA on smartwatches, microinteractions, and μEMA.

### 2.1 Engagement with EMA

The number of questions presented per prompt varies between and within EMA studies [56, 94]. When prompted, participants are asked about their experiences in the moment or recent past (e.g., “Right now/In the past hour, how stressed do/did you feel?”); the questions can be answered via single-answer selection (e.g., Likert scales), text entry, or a sliding analog scale [86]. A best practice for EMA is to minimize this burden by keeping the interactions “simple and unobtrusive” [18]. The question sets used with EMA are, however, typically modifications of paper-based surveys and not necessarily optimized for in-situ answering [18]. For example, a recent study found that longer EMA surveys increase perceived burden and reduce data quality, regardless of prompting frequency [22]. Cognitively complex questions can also induce burden, impacting EMA data quality [82].

The burden of EMA can accumulate, impacting compliance and data quality [7, 87]. Even in a 1–2-week study, compliance drops [80] and “careless response behavior” may be observed [93]. Although reducing survey frequency might control the burden somewhat, high frequency is desired for acceptable ecological validity [70]. However, a meta-review by Wrzus and Neubauer highlighted how researchers have traditionally kept the study duration shorter for more frequent assessments [95]. Nevetheless, in another meta-review, Kawashima et. al. [39] reported that studies using EMA are increasingly collecting data at a higher frequency, for longer durations, and are raising the bar for acceptable response rate to maintain participation. EMA designs must balance data frequency and user burden [18].

A common approach to boost engagement with EMA is offering compensation based on response rates, but this approach can be costly for large-scale, longitudinal studies. Thus, alternative ways of improving engagement with EMA are needed. For instance, Truong et al. developed a smartphone lock-screen-based method to obtain responses to EMA questions when users unlock their phones [84]; this method has been used to measure mood instability in real-world settings [71]. The researchers, however, limited the frequency with which EMAs were shown on the lock screen to avoid burden and minimize careless responding. Similarly, Van Berkel et al. developed gamified EMA where participants rated ascending keywords (like Tetris) as relevant/irrelevant to their current location within a time limit [6]. Their three-week study showed that the gamified EMA yielded higher response rates than the non-gamified EMA. Dejonckheere et al., however, showed that while gamifying EMA improved response rates, it hurt response quality by encouraging careless responding to ‘game’ the protocol [17]. In this case, Kleiman et. al.’s work is an exception as gamification showed improved engagement but without compromising response quality [46]. Alternatively, researchers have found that using response visualizations as feedback during experience sampling improves compliance [31], but this approach has not been tested longitudinally, and novelty may wear off, impacting data quality.

### 2.2 EMA Deployed on Smartwatches

Wrist-worn smartwatches can be accessed more easily than smartphones and thus have been used to gather real-time self-reports. For instance, Kikuchi et al. used watches to prompt participants several times a day with questions on tension-type headaches, depressive mood, and fatigue [42, 43]. Timmerman et al. adapted the single-item perceived exertion scale to the Apple Watch screen [83]. Similarly, Dai et al. and Laborde et al. used the Android watch’s circular touchscreen dial to display a 10-point numeric pain rating scale [16, 50]. However, these studies used paper-based surveys adjusted for the watch screen; the cognitive complexity of the questions was equivalent to an EMA question.

Researchers have also explored the design challenges inherent in using smartwatches for EMA. For instance, Hernandez et al. compared EMA delivery on a smartwatch and a smartphone and showed no statistically significant difference in their response rates [27]. Likewise, Yan et al. explored different interaction patterns (e.g., gesture input and sliding on numeric rating scales) to assess the perceived burden of self-reporting using smartwatches [97, 98], but at a lower temporal density than many EMA studies. In similar research, Markopolous et al. [54] found that bezel-based interactions on smartwatches yield slower response times and produce more accurate self-reports than swipe-based interactions. While smartwatches are promising devices to get users’ attention, directly implementing smartphone EMA on smartwatches has limitations. First, the limited screen real estate on the watch prevents seamless interaction with sensitive scales (e.g., a five-point Likert scale does not fit well on the watch). Second, smartwatches are not designed for prolonged interaction, making them burdensome for longer EMA surveys.

### 2.3 Wearable Microinteractions

Microinteractions are short interactions that take only 3–4 seconds to complete [4] and reduce the “gulf of execution” (i.e., the user-input time) in interfaces [32, 61]. Common examples of microinteractions include turning lights on, unlocking a smartphone, and checking the time on a watch — tasks we perform numerous times a day without deliberation. Smartwatches, among other wearables, enable microinteractions because they can be accessed quickly. Small screen real estate enables smartwatch interfaces to be deliberately designed for tasks that are meant to be completed with a quick glanceable interaction [64]. Smartwatches are most frequently used to check time and notifications — 3–4 second microinteractions [64]. To enable these microinteractions, smartwatch interfaces must be minimalistic, displaying only relevant actions for that moment [2]. If designed well, smartwatch use may more seamlessly mesh with our routines than smartphone use, and smartphones may be more likely to become distracting [10, 41, 89].

### 2.4 μEMA on Smartwatches

Microinteraction-EMA (μEMA) is a type of EMA where each prompt presents only one single question that can be answered on a smartwatch with a glance and tap microinteraction, taking only 3–4 seconds [33]. The microinteraction is achievable when four criteria are met: 1) fast, easy access to the smartwatch with a flick of the wrist, 2) a cognitively simple question, 3) a question that is answerable with a single-tap rapid response, and 4) each prompt being guaranteed to be followed by only one question. The design goal of a μEMA question is to avoid scrolling on the watch screen (with yes/no type answers) — a fundamentally different approach from simply putting EMA questions on smartwatches without adaptation. Prior work has shown that these yes/no type questions in traditional surveys yield higher response rates than other response formats [69].

In four-week-long studies, μEMA response rates have been higher than smartphone EMA and smartwatch EMA (i.e., EMA on a smartwatch), despite four times the amount of prompting with μEMA [33, 65]. A one-week study with six μEMA prompts an hour (i.e., 72 times a day for 12 hours) measuring physical activity also found strong criterion validity of μEMA responses compared to a high-sampling-rate research-grade accelerometer [67]. Similarly, researchers found support for data validity when comparing self-reported stress through μEMA and physiological sensors in a lab setting [44]. So far, μEMA has been used to measure hyperarousal [51], stress [44], indoor comfort [35], alcohol use [48, 78], audio experiences [96], and the method has been adapted to capture self-report using pervasive displays [63]. In addition, μEMA with voice input [1, 52] has been explored with applications for persons with aphasia [28].

μEMA has also been found to yield reliable response rates across most user contexts (e.g., different times of day, locations, and days of the week) where EMA has historically led to missing data [66]. One limitation of μEMA is that each prompt can only trigger *only* one question measuring *only* one construct with a binary response set. Thus, μEMA yields less information per prompt and question than EMA. However, this binary response set enables μEMA to collect data at a higher frequency without compromising the burden. In this paper, we evaluate whether μEMA is a viable method for personalized data collection in longitudinal studies that last for many months or years.

## 3 METHODOLOGY

We used data collected as part of the Temporal Influences in Movements and Exercise (TIME^1^) study — a study to examine temporal factors that influence behavior adoption and maintenance of physical activity, sedentary behavior, and sleep [68, 92]. Data were collected via a smartphone/smartwatch app for up to one year using 1) passive sensors on the smartphones and smartwatches, 2) smartphone-based EMA, and 3) smartwatch-based μEMA. Data collection took place nationally across the US from May 2020 to June 2022. The details of the TIME study design are beyond the scope of this paper and are available in prior work [68, 92]. In this paper, we provide only study design information relevant to the research questions on user engagement.

### 3.1 Study Design

**Fig. 1.**
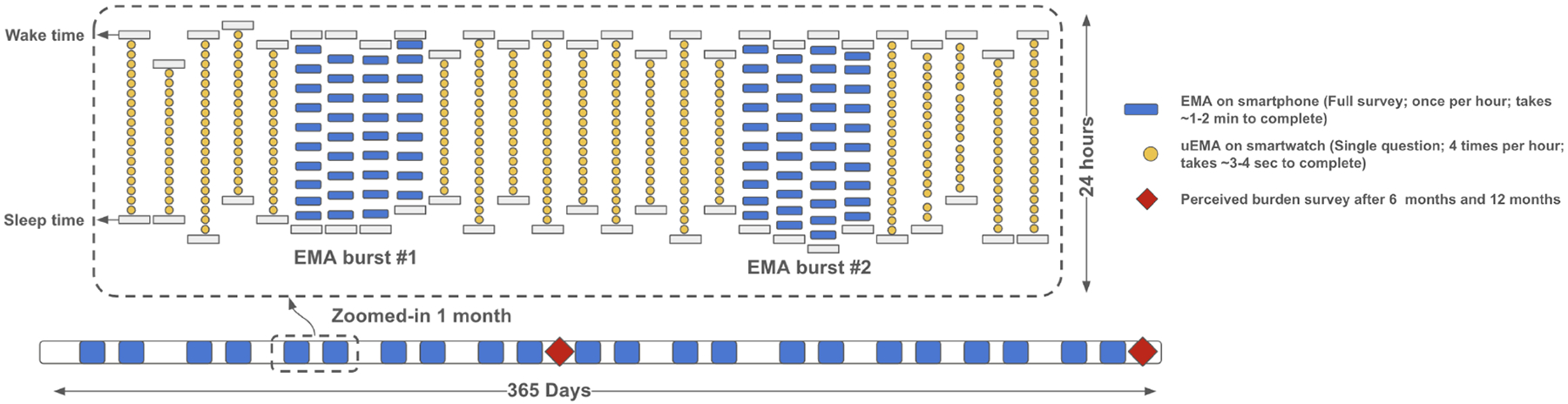
Schematic diagram of TIME study for a participant who completed μEMA, EMA, and perceived burden surveys for 12 months. One month of data collection is zoomed in for illustrative purposes. EMA was prompted in four-day measurement bursts (one prompt/hour) and μEMA was presented on non-burst days (four prompts/hour) between participants’ self-reported waking periods.

The study consisted of nested measurement bursts of EMA that occurred twice a month with 8–12 days between bursts (Figure 1). Each burst period was four days long, including the two weekend days. The remaining 22–23 days per month were reserved for μEMA prompting. The app automatically set the study schedule and controlled the smartwatch so that μEMA was not prompted on EMA burst days. Thus, for a participant in the study for 12 months, there were 270 μEMA and 90 EMA burst days (Table 1). The TIME study uses burst-based design for two reasons: 1) Continuous EMA data collection over long periods can be burdensome, leading to poor data quality, and sampling for intensive short periods occasionally is a strategy to avoid burden accumulation [9] and 2) burst designs effectively capture temporal dynamics both within short time windows (during bursts) and across longer periods (between bursts) [25].

There were 11 affect-based constructs common to both μEMA and EMA for which participants provided self-report data. These constructs include stress, sadness, happiness, fatigue, frustration, focus, routine, tension, nervousness, control, and relaxation. Participants also completed a user burden survey [79] via RedCap [26] after six and 12 months.

### 3.2 μEMA Design Overview

**Fig. 2.**
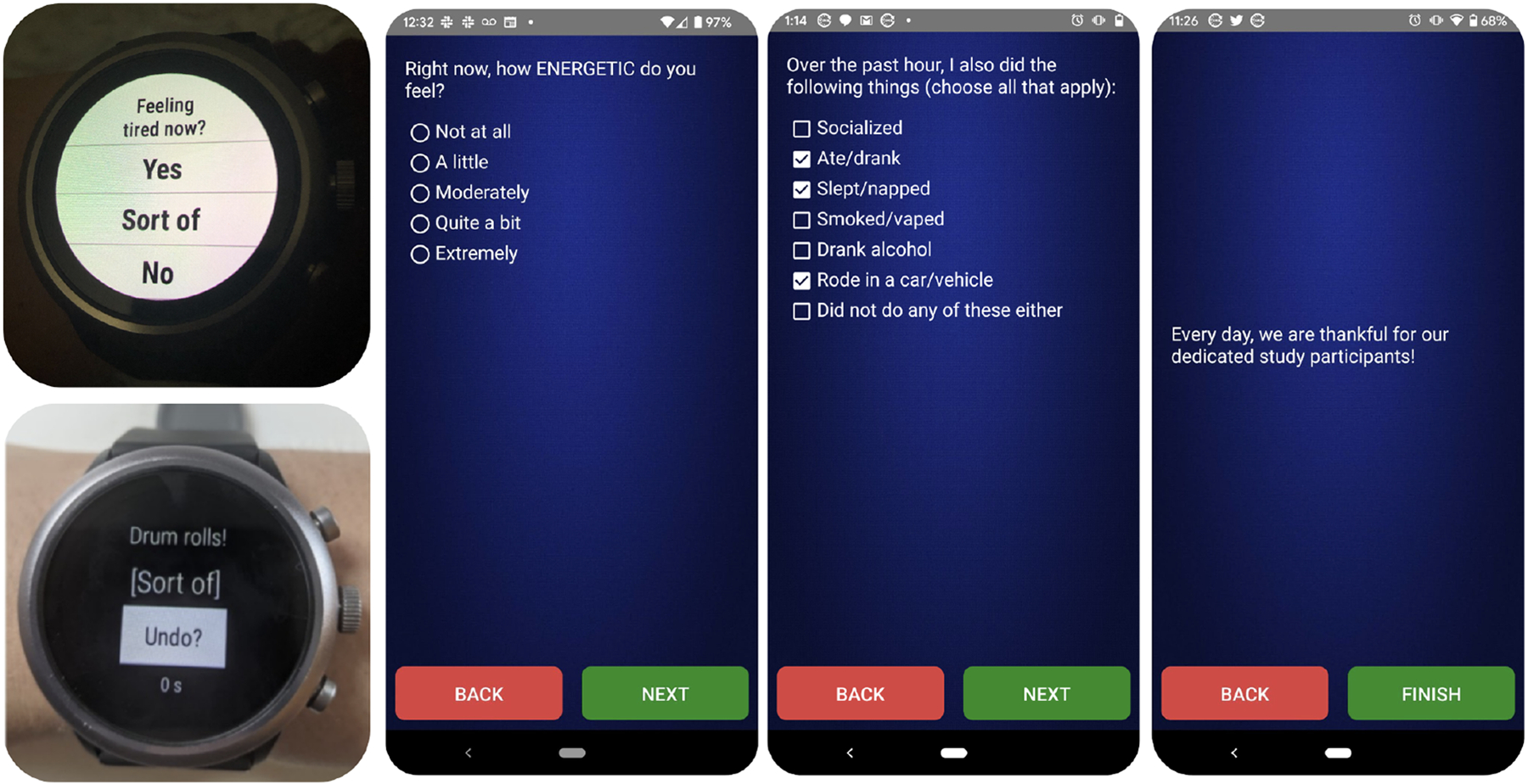
(Left) μEMA prompt (on Fossil Gen 4) with only one question on the smartwatch and an undo screen to change response within 3 s (with an acknowledgment message). (Right) EMA questions on a smartphone with five answer options and seven multiple-selection options (on Moto G5). Each EMA prompt has 18+ such questions. Also shown is an example acknowledgment message presented after answering all the questions in an EMA survey.

The μEMA app was built for Android Wear OS 2.0. Each prompt (Figure 2, left) started with a six-second vibration and presented *only* one question (e.g, Feeling stressed now?) with “Yes/Sort of/No” options. After the prompt, the participant had 20 seconds to tap on an answer. If the participant did not respond in 20s, the prompt was closed, and a missed response was logged. If the participant tapped on an answer within 20 s, a second screen displayed the selected answer with a button to ‘Undo’ within 3 s. If the participant decided to undo, the original question was presented again, or the prompt was closed, and the original answer was logged. Users could use undo only once. The undo screen also displayed a “thank you” message that changed for each prompt (Figure 2, left).

μEMA prompts occurred four times per hour with at least an eight-minute gap between two prompts. The μEMA prompting started 15 min after the self-reported wake time and stopped 15 minutes before the self-reported sleep time [68]. Thus, in a typical waking day of 16 h (assuming 8 h of sleep), μEMA prompted 62 times. The watch could be set to do-not-disturb continuously for one hour to pause prompting; after which, the app notified the user to turn off do-not-disturb to prevent further data loss. Thirty different types of μEMA questions measured behavioral, psychological, and contextual constructs (e.g., “Feeling fatigued?” or “Feeling happy?” - “Yes/ Sort of/ No”). With only one question per prompt, 90% of the prompts within a day were reserved for such construct questions, and the remaining 10% were reserved for validation questions (e.g., “Do pigs fly?” or “1 + 3 = 5?” – “Yes/ No”). Validation questions had an unambiguous correct answer that could be used to check participants’ careless responses after the study. Thus, if a participant collects data on all 270 days, with 62 μEMA prompts per day, we can expect 16,740 interruptions for that participant, but each of those interruptions is only a single question that can be answered with a glanceable microinteraction (i.e., interrupt more, ask less).

### 3.3 EMA Design Overview

The EMA app was built for Android OS version 7+. The smartphone prompted with a vibration (11 s) and/or audio chime (3 s) and presented the first question (with 5+ answers) of the EMA question set. For instance, to measure stress, the EMA question was framed as “Right now, how stressed do you feel? - Extremely/ Quite a bit/ Moderately/ A Little/ Not at all.” Each EMA survey presented 18–23 questions one by one. Participants could also return, and change the answers to previous questions (Figure 2, right).

When prompted, the participant had 10 min to respond to the EMA. If the survey was not completed within 10 min, it was re-prompted after an additional 5 min. If all the questions were answered, then the survey was recorded as completed. If only some of the questions were answered, the survey was recorded as partially completed, and if none of the questions were answered, then the survey was recorded as missed. During the EMA burst days, prompting started 30 min after self-reported wake time and stopped 30 min before self-reported sleep time and could be paused using do-not-disturb mode on the phone for one hour [92]. EMA surveys included questions on positive-negative affect, physical activity, sedentary time, and sleep. At the end of each survey, participants were presented with a unique thank you message that changed each time (Figure 2, right). When notified about an upcoming burst period, participants could delay the burst period for only up to two days. Thus, assuming the same 16h waking period, a participant can expect 15 EMA interruptions on a given day. Thus, if the participant completes all the 90 days of EMA data collection, the participant can expect 1,440 EMA interruptions in the study, but each of those interruptions presents multiple questions back-to-back (i.e., interrupt less, ask more).

### 3.4 Participant Recruitment and Procedures

Participants were eligible for the study if they 1) owned a smartphone with Android 6+ as their only personal phone with no intention to switch to a non-Android phone, 2) did not wear a smartwatch already, 3) were between 18–29 years old living in the US, 4) engaged in recommended levels of physical activity (or intended to in the next 12 months), 5) spoke and read English, 6) had Wi-Fi connectivity, 7) did not have any physical or cognitive limitations that prevented participation, and 8) could wear a smartwatch and answer smartphone/smartwatch surveys. All the procedures were approved by the Institutional Review Board (IRB) at the Univ. of Southern California (USC). Participants were recruited via 1) emails to individuals enrolled in prior IRB-approved studies, 2) flyers in the Los Angeles Metropolitan Area, and 3) ResearchMatch [22] — N = 250 participants were recruited.

Study onboarding and all participant interactions were conducted remotely. After screening, research staff individually met with each participant via Zoom to obtain informed consent. Staff then guided the participants through the app installation on their smartphones. Researchers monitored EMA compliance for the first four-day EMA burst. If the compliance for the first EMA burst was less than eight surveys/day on all days, participants were withdrawn from the study, otherwise, they could continue in the study and were mailed a smartwatch (Fossil Gen 4 or Gen 5). After receiving the watch, participants scheduled a remote setup session with research staff to configure the smartwatch and phone. This supervised process ensured consistent settings across devices for uninterrupted data collection [68]. Consequently, the first few non-burst days in the TIME study did not include μEMA data collection. Accounting for the initial burst, watch shipment delays, and setup, μEMA initiation took a median of 15 days (IQR: 13, 18) [68].

Participants were asked to wear the watch for 23 hours/day and charge it daily. At the end of each month, participants were compensated up to $100 for EMA completion. Participants received $20 for wearing the smartwatch for 23 hours on at least 24 days/month. In addition, if participants had more than 50% completion with μEMA at the end of the study, they could keep the watch permanently. However, no monthly compensations were associated with μEMA completion.

### 3.5 Data Monitoring and Quality Checking

The research team monitored participant EMA completion and smartwatch wear time every month. In case of malfunctioning watches because of Wear OS updates or faulty hardware, the app was updated remotely, or a replacement watch was mailed. Participants were not penalized for this missing data. The research team logged issues that might impact compliance as participants brought them up and manually flagged these dates to be removed from the analysis. Based on this study’s dataset, we divided participants into three groups in this paper -*completed*, *withdrew*, and *unenrolled*.

#### 3.5.1 Completed Participants.

*Completed* participants responded to both EMA and μEMA for a full one-year period in the TIME study. Study completion is only determined based on whether participants completed all the EMA bursts, regardless of how they performed with μEMA data collection. *Completed* participants answered the user burden survey both in the sixth and twelfth months of the study.

#### 3.5.2 Withdrew Participants.

*Withdrew* participants voluntarily withdrew from the study after six months of data collection, citing various reasons including feeling burdened answering EMA surveys, lifestyle changes (such as a change in job that did not permit phone usage), and change of device (to an iOS device), among others.

#### 3.5.3 Unenrolled Participants.

*Unenrolled* participants are those who research staff removed from the study due to poor EMA response rates. Note that participants were only unenrolled for their poor performance with EMA. μEMA performance did not impact the participant’s continued enrollment in the study.

## 4 RESULTS

We compared user engagement for the three participant groups – *completed*, *withdrew*, and *unenrolled*, and measured user engagement in terms of response rates and perceived burden.

### 4.1 Participants

We *only* included those participants in our analysis who completed at least six months of data collection in TIME study, resulting in data from 179 participants. This ensured that we have 1) at least six months of EMA and μEMA data across participants (a reasonably long duration compared to many prior EMA studies [95]), 2) participants’ responses to at least one user burden survey administered after six months into study (measuring both response rate and burden components of user engagement), and 3) a comparison of μEMA’s engagement with EMA under practical intensive longitudinal settings (goal of this analysis).

We cleaned the data in two steps. First, we removed days when the prompts were not delivered because of a phone or watch malfunction (Section 3.5). The common reasons for EMA data removal were smartphone replacement and Android updates that restricted background processes. The common reasons for μEMA data removal were watch damage, low memory due to “bloatware”, and Wear OS getting stuck in the boot loop. Data from those days were removed because data loss was out of participants’ control.

Second, we also check μEMA validation rate (total correct validation responses / total validation questions delivered) for all the participants. Overall, the average validation rate was 93.53% (SD = 23.00%). However, we identified two participants (P1 and P2) whose accuracy on validation questions was below the 1.5 interquartile range (54% and 58%). P1 indicated in a compliance check follow-up that questions on the watch and phone were answered carelessly. P2 had an unresponsive smartwatch that did not register taps consistently but never reported this problem to the research staff. Due to these reasons (obtained qualitatively), we removed P1 and P2 from the analysis, leaving 177 remaining participants for the analysis. Among them, 135 participants completed the study, 23 voluntarily withdrew from the study, and 19 were unenrolled by the research staff (Table 2).

### 4.2 Response Rates

**Fig. 3.**
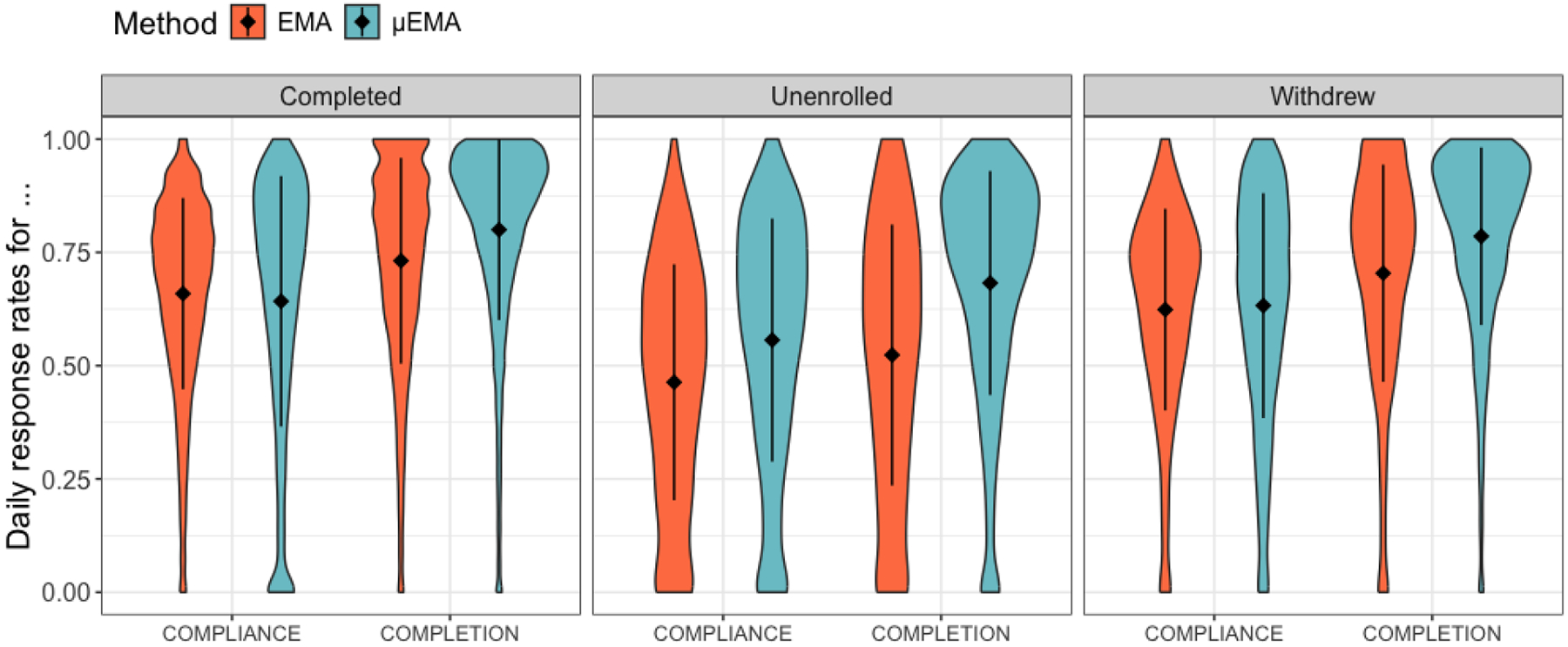
Compliance and completion rates (with mean) of μEMA and EMA for *completed*, *withdrew*, and *unenrolled* participants.

We measured response rates as the total number of prompts answered out of the total delivered and/or scheduled (expected) prompts. Across 179 participants, we had 16,597 EMA burst days and 36,311 μEMA days, after removing outlier participants and days with missing data caused by device malfunctioning (Section 3.5). We expected 233,903 EMA prompts during the burst periods; 210,597 of these were delivered, and 148,931 were completed. For μEMA, we expected 2,153,362 prompts, of which 1,706,324 were delivered and 1,365,712 were completed. We measured response rates as compliance and completion rates based on prior studies [33, 56, 65, 67] (Figure 3 and Table 3).

#### 4.2.1 Compliance Rate.

The compliance rate is the number of prompts answered out of the total number of prompts expected to be delivered, accounting for all the data loss due to the watch/phone being off or in do-not-disturb mode. Thus, Compliance Rate = Prompts Answered / Prompts Scheduled. Because the broader goals of the TIME study involve modeling health behaviors using self-report and passive sensor data, the compliance rate here penalized participants for the instances when devices were switched off (causing both self-report and sensor data loss). Note that scheduled prompts do not include prompts not triggered due to device malfunctioning or missing data outside the participants’ control (Section 3.5). A prompt is for only one question for μEMA, and a question set for EMA. An EMA prompt is considered completed when all the questions in a question set are answered. Overall, the compliance rate of EMA was 63.7% and the compliance rate for μEMA was 63.4% (Table 3, Figure 3).

#### 4.2.2 Completion Rate.

Completion rate is the number of prompts answered out of the total number of prompts delivered, not accounting for all the data loss due to the watch/phone being off or in do-not-disturb. Thus, Completion Rate = Prompts Answered / Prompts Delivered. Overall, the completion rate for EMA was 70.7% and the completion rate for μEMA was 80.0% (Table 3; Figure 3).

EMA literature has inconsistencies in reporting response rates [62]. To address this, we compute both compliance and completion rates. For example, if a participant is expected to receive 10 prompts but only receives five due to the device being off and answers all five, the compliance rate is 50%, but the completion rate is 100%. Completion rates are always equal to or higher than compliance rates and can inflate response rates if data loss due to device issues is not considered. As a result, the compliance rate helps provide a complete picture of participants’ response rates.

### 4.3 μEMA vs. EMA Response Rates

To compare μEMA and EMA response rates, we used each study day as the unit of analysis (that is, day-level outcomes nested within participants). During the TIME study, participants completed surveys via EMA on burst days or μEMA on nonburst days. Also, at the day level, we can measure both the number of originally scheduled prompts (for the compliance rate) and the number of actually delivered prompts (for completion rate).

First, to compare μEMA and EMA compliance rates, we use each prompting day (μEMA or EMA day) as the unit of analysis and treat EMA type (i.e., μEMA vs. EMA) and participant status (i.e., *completed*, *withdrew*, or *unenrolled*) as regressors, along with an interaction term between EMA type and participant status. We dummy code participant status and EMA type with *Completed* and ‘EMA’ as the reference categories. Finally, we model the probability of completing the expected prompts within a day (the binary outcome is 1 if completed all the delivered prompts or else 0) using a mixed-effects logistic regression (using the lme4 package [5]). We chose this model because 1) it accounts for the lack of independence among repeated observations nested within participants, and 2) it helps model the log-odds of completing the scheduled/expected prompts. Thus, this model has two levels - day-level (level-1) and participant-level (level-2):

(1) $$\text{logit}\left(\theta_{\text{ij}}\right)=\beta_{0}+\beta_{1}\left(\mu \text{EM}{\text{A}}_{\text{ij}}\right)+\beta_{2}\left(\text{Unenrolle}{\text{d}}_{i}\right)+\beta_{3}\left(\text{Withdre}{\text{w}}_{i}\right)+\;\beta_{4}\left(\mu \text{EM}{\text{A}}_{\text{ij}}\right)*\left(\text{Unenrolle}{\text{d}}_{i}\right)+\beta_{5}\left(\mu \text{EM}{\text{A}}_{\text{ij}}\right)*\left(\text{Withdre}{\text{w}}_{i}\right)+u_{i}$$

Here, the *θ*_ij_ is the odds of completing the scheduled prompts for participant i on day j. The *β*_0_ is fixed-effects intercept, μ*EMA*_ij_ denotes the day type for participant i on day j (level-1; μEMA = 1 and EMA = 0), *Unenrolled*_i_ and *Withdrew*_i_ are the participant i’s status at the end of the study (For completed participants *Unenrolled*_i_ = 0 and *Withdrew*_i_ = 0), and *u*_i_ is the random intercept for participant i (level-2) to account for lack of independence within a participant with repeated observations. As a result, the final model fit (at p < 0.001) for compliance rate was (Table 4):

(2) $$\text{logit}\left(\theta_{\text{ij}}\right)=0.70-0.11\left(\mu \text{EM}{\text{A}}_{\text{ij}}\right)-0.81\left(\text{Unenrolle}{\text{d}}_{i}\right)-0.17\left(\text{Withdre}{\text{w}}_{i}\right)+\;0.52\left(\mu \text{EM}{\text{A}}_{\text{ij}}\right)*\left(\text{Unenrolle}{\text{d}}_{i}\right)+0.15\left(\mu \text{EM}{\text{A}}_{\text{ij}}\right)*\left(\text{Withdre}{\text{w}}_{i}\right)+u_{i}$$

We observed a statistically significant interaction between participant status and the EMA type to predict the probability of answering the scheduled prompts on a given day. Compared to answering the scheduled EMA prompts, *completed* participants were 0.89 (*e*^−0.11^, CI: 0.88, 0.90) times more likely to answer scheduled μEMA prompts, participants who *withdrew* were 1.04 (*e*^−0.11^*e^0.52^, CI: 0.99, 1.08) times more likely to answer scheduled μEMA prompts, and *unenrolled* participants were 1.50 (*e*^−0.11^*e^0.15^, CI:1.43,1.58) times more likely to answer scheduled μEMA prompts (Table 5).

Second, to compare μEMA and EMA completion rates, we model the probability of completing the delivered prompts within a day using the same mixed-effects logistic regression as above, but where *θ*_ij_ is the odds of completing a delivered prompt for participant i on day j. The final model fit (at p<0.001) for the completion rate was (Table 4):

(3) $$\text{logit}\left(\theta_{\text{ij}}\right)=1.13+0.42\left(\mu \text{EM}{\text{A}}_{\text{ij}}\right)-0.96\left(\text{Unenrolle}{\text{d}}_{i}\right)-0.19\left(\text{Withdre}{\text{w}}_{i}\right)+\;0.38\left(\mu \text{EM}{\text{A}}_{\text{ij}}\right)*\left(\text{Unenrolle}{\text{d}}_{i}\right)+0.09\left(\mu \text{EM}{\text{A}}_{\text{ij}}\right)*\left(\text{Withdre}{\text{w}}_{i}\right)+u_{i}$$

Thus, compared to answering the delivered EMA prompts, *completed* participants were 1.53 (CI:1.51, 1.54) times more likely to answer delivered μEMA prompts, participants who *withdrew* were 1.67 (CI:1.59, 1.74) times more likely to answer delivered μEMA prompts, and *unenrolled* participants were 2.25 (CI: 2.13, 2.34) times more likely to answer delivered μEMA prompts (Table 5). For both compliance and completion rate models, we also included age and sex (Table 2) as level-2 covariates. However, we did not observe any statistically significant impact of sociodemographics on response rates. We report other socioeconomic variables (e.g., education and employment) in Table 10 (Appendix A).

### 4.4 μEMA vs. EMA Perceived Burden

**Fig. 4.**
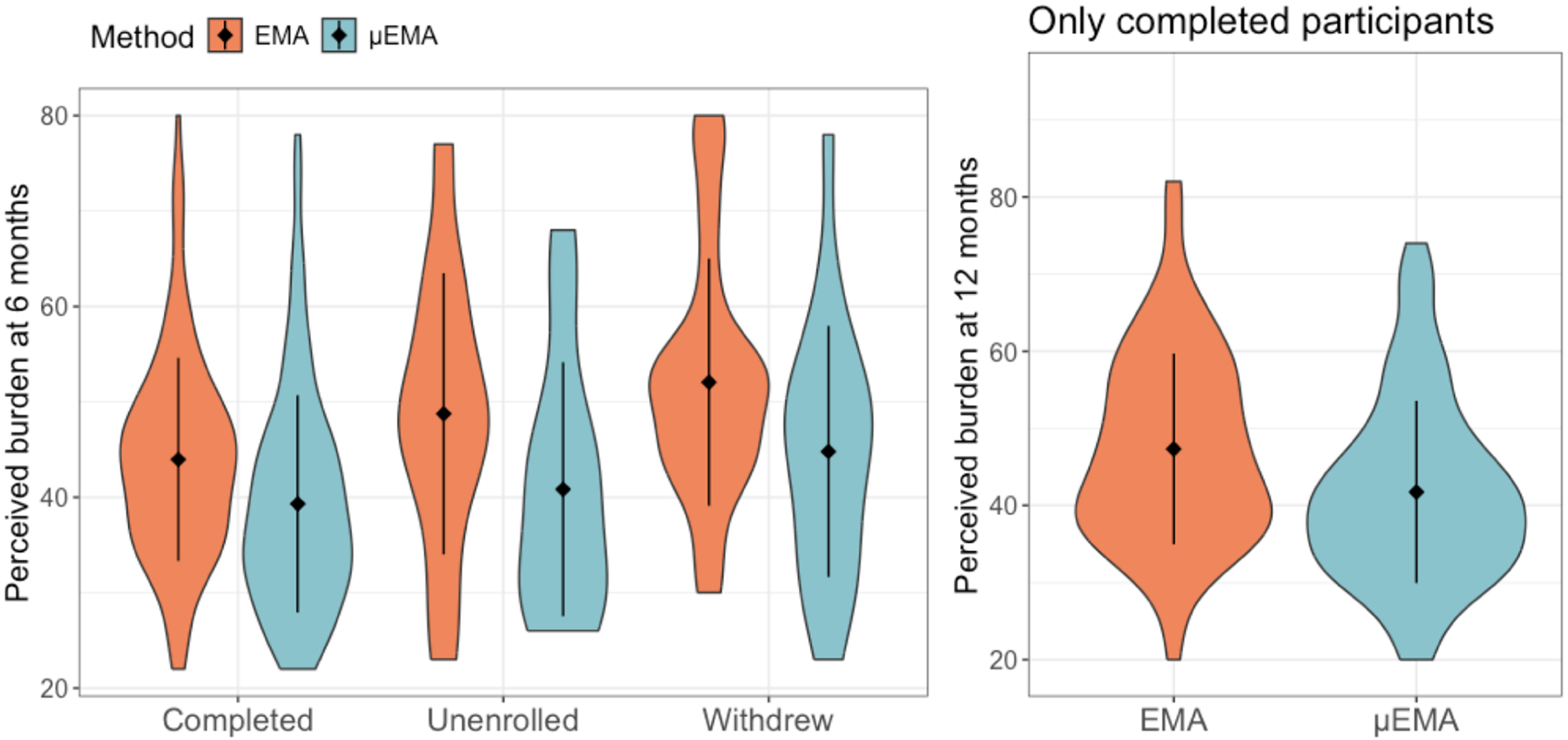
(Left) Perceived burden measured after six months across *completed*, *unenrolled*, and *withdrew* participants. No statistically significant interaction effects were observed between participant status and EMA type (μEMA vs. EMA). (Right) Perceived burden was measured after 12 months for only *completed* participants.

To address RQ2, we measured the perceived burden with EMA and μEMA using the user burden scale [79]. The original scale consisted of six subscales including ease-of-use (four questions (Qs), physical burden (two Qs), time/social burden (four Qs), cognitive burden (five Qs), privacy burden(three Qs), and financial burden (two Qs). Each question uses a five-point Likert scale. We excluded the privacy and financial burden subscales because participants 1) consented to collect this data for research with data encryption, and 2) were paid to provide EMA data and did not incur any costs. We then added two questions on interruption burden (from prior work [33] and built on the work by [7, 57], Table 8, Appendix A) resulting in 19 questions, and the perceived burden ranged from 19 (least burdensome) to 95 (most burdensome). We compared perceived burden after six months and 12 months in the study (Table 6 and Figure 4).

#### 4.4.1 μEMA vs. EMA Perceived Burden After Six Months.

After six months, we had 177 participants active in the study who later either *completed* the study, *withdrew* voluntarily, or were *unenrolled* by the research staff. When measuring perceived burden after six months, the mean perceived burden for EMA among *completed* participants was 43.96 (SD = 10.65), 52.05 (SD = 12.96) among those who *withdrew*, and 48.75 (SD = 14.73) for unenrolled participants. Whereas, the mean perceived burden for μEMA among *completed* participants was 39.31 (SD = 11.39), 44.79 (SD = 13.17) among those who *withdrawn*, and 40.83 (SD = 13.32) among *unenrolled* participants. We assessed if the burden measured after six months was associated with participants’ status at the end of the study. We used two-way ANOVA with perceived burden as the outcome and the EMA type (μEMA vs. EMA) and participant status as predictors (described below).


(4) $$\text{PerceiveBurde}n_{\text{ij}}=\mu +\mu \text{EM}{\text{A}}_{i}+\text{Statu}s_{j}+{\left(\mu \text{EMA}*\text{Status}\right)}_{\text{ij}}+\epsilon_{\text{ij}}$$

Here, *PerceiveBurden*_ij_ is perceived burden observation for the *i*^th^ level of μEMA (where μEMA = 0 or 1) and *j*^th^ level of participant status (*completed*, *withdrew*, or *unenrolled*), and *ϵ*_ij_ is the random error. We observed no statistically significant interaction between EMA type and participant status. However, there were statistically significant main effects with the moderate effect of EMA type (F = 9.84, p <0.01, partial-*η*^2^ = 0.05) and participant status (F = 4.61, p <0.01, partial-*η*^2^ = 0.05). Moreover, post-hoc pairwise comparison using Tukey adjustment showed that participants perceived μEMA to be less burdensome than EMA. Similarly, pairwise comparison revealed statistically significant differences in perceived burden between *withdrew* and *completed* participants, where those who *withdrew* perceived higher burden than *completed* participants (Figure 4 (left) and Table 7).

#### 4.4.2 μEMA vs. EMA Perceived Burden After 12 Months.

To compare the perceived burden after 12 months (i.e., only *completed* participants), we used the paired-sample t-test. We observed that participants perceived μEMA to be less burdensome than EMA (t = 6.3, p <0.001, 95% CI: 3.83, 7.34) with a moderate effect (Cohen’s d = 0.46) despite similar response rates (Table 6 and Figure 4 (Right)).

### 4.5 μEMA vs. EMA Response Comparison

To support the results for RQ1 and RQ2, we conducted further analysis to examine how correlated the data collected from EMA and μEMA are. Because μEMA and EMA collected data on completely different days, a direct comparison between the prompts or interruptions for each construct is not possible. We can, however, assess whether μEMA and EMA variability are related across the 11 common constructs (e.g., for a given participant, how related are the variabilities captured by μEMA and EMA?). This approach is consistent with prior work validating the Day Reconstruction Method (DRM) by using variance in fatigue between DRM and self-report surveys[37]. Hence, we computed correlations between the within-participant variances (variance at the participant-level for the entire duration of data collection) captured by μEMA and EMA across the 11 constructs (stress, sadness, frustration, focus, tension, happiness, relaxation, nervousness, routine, fatigue, and control).

**Fig. 5.**
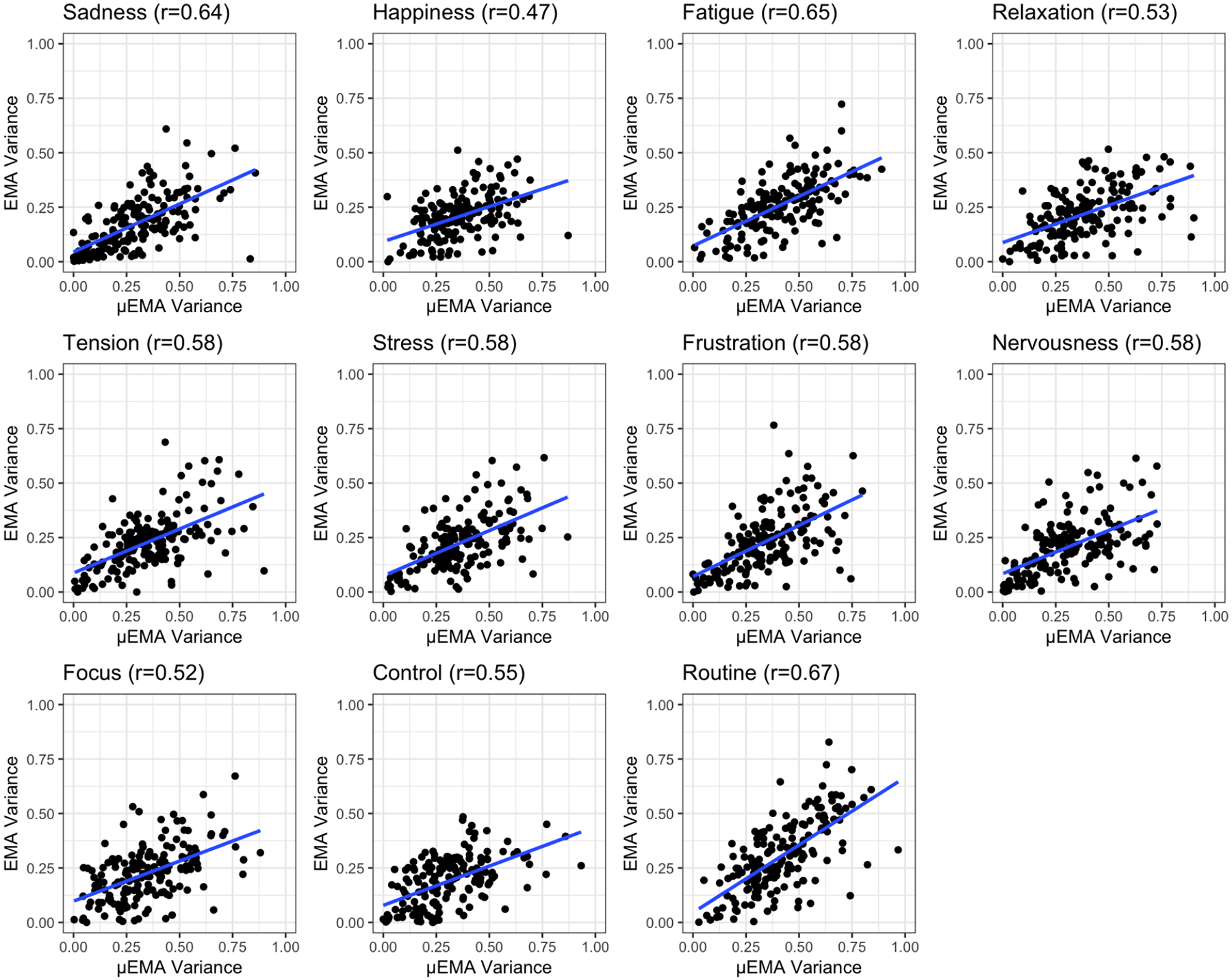
Correlation plots comparing μEMA and EMA within-person variances across 11 constructs.

We first converted the 5-point EMA scale to the 3-point μEMA scale for consistency in measurements [36]. Specifically, the EMA answers “Extremely” and “Quite a bit” were mapped to “Yes” (scored as 3), “Moderately” and “A little” were mapped to “Maybe” (scored as 2), and “Not at all” was mapped to “No” (scored as 1) in μEMA. Next, for each participant (N = 177), we calculated the within-participant variance for each construct for both μEMA and EMA. Finally, we computed the Pearson correlation (r) across all constructs to compare within-participant variances between μEMA and EMA. Overall, we observed moderate to strong correlations between μEMA and EMA within-participant variances (0.46 ≤ *r* ≤ 0.67, *p* < 0.001) across all constructs. Figure 5 shows the correlation plots (with the best-fit line) for all 11 constructs. For completeness, we also conducted the same analysis between the 5-point EMA and μEMA scales, and the results were similar to those of the 3-point EMA (Table 9, Appendix A). However, for consistency, we report only the results using the 3-point EMA scale.

## 5 DISCUSSION

μEMA is designed to capture self-reports at high frequency with single-item surveys that can be answered with quick microinteractions. While prior studies have shown μEMA to be sustainable in shorter durations (e.g., 1 to 4 weeks period), we conducted the first large-scale empirical study comparing μEMA and EMA in an intensive longitudinal data collection lasting one year. This research aims not to replace EMA with μEMA, but to complement both methods in intensive longitudinal data collection (e.g., μEMA collecting data when users are least receptive to EMA). We want to learn instances where μEMA could be complementary to the existing EMA methodology. Thus, using data from the TIME study, we assessed μEMA for two engagement metrics — response rates and perceived burden. The response rate and perceived burden together help capture a fundamental trade-off in intensive longitudinal data collection - i.e., “interrupt more, ask less” (μEMA) vs “interrupt less, ask more” (EMA). To identify user segments that μEMA is well-suited for, we divided the TIME study participants into three categories – *completed*, *withdrew*, and *unenrolled* participants, based on their EMA response rates. In this section, we delve deeper into two engagement patterns: 1) μEMA yields higher response rates for participants who performed poorly with EMA, and 2) μEMA is perceived as less burdensome than EMA regardless of participants’ response rates.

### 5.1 μEMA Yields Higher Response Rates Even When EMA Is Unsustainable

μEMA’s high response rate were higher than EMA, *even though* the TIME study was biased to favor EMA in several ways. First, in longitudinal data collection, more prompting is expected to create more user burden [85]. In the TIME study, participants were exposed to more μEMA interruptions per day than EMA (during the EMA days). Second, they were exposed to μEMA interruptions on more days ( 270 days for μEMA vs. 90 days for EMA). Thus, in terms of the absolute number of prompts, μEMA was 12 times as interruptive as EMA. Third, participants were financially compensated for their participation in EMA. Prior meta-review shows that financial incentives significantly drive engagement with EMA [95]. Finally, Research staff regularly checked with participants to encourage them to complete the EMA prompts. Participants were unenrolled according to their engagement with EMA and were considered *completed* only if they yielded reasonable response rates from the EMA.

**Fig. 6.**
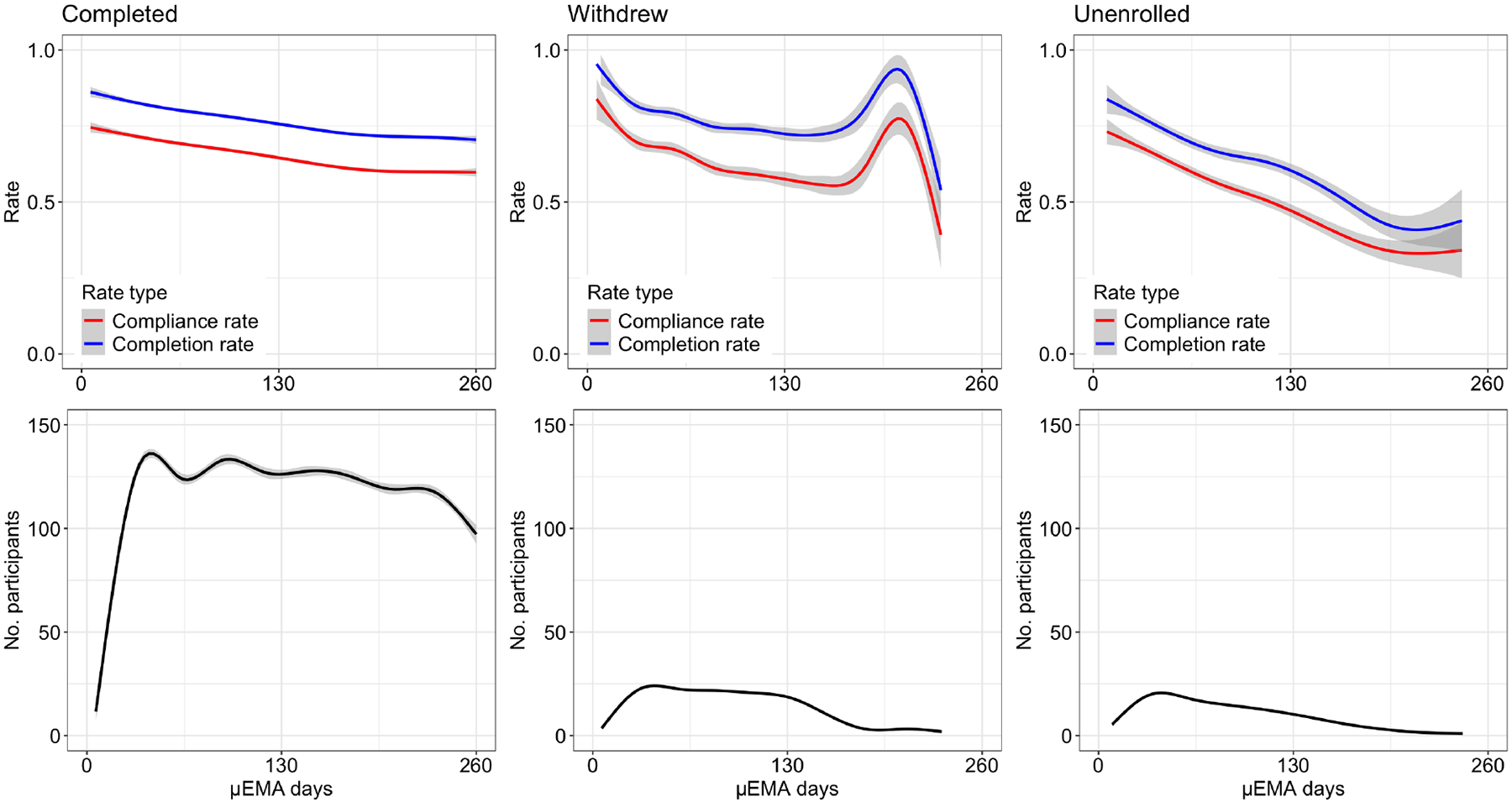
Daily trends in μEMA compliance and completion rates for *completed*, *withdrew*, and *unenrolled* participants. μEMA data collection takes place over 260 days making the 130th day the sixth month of data collection in TIME study. NOTE: The μEMA data collection starts on 15th non-burst day

Despite these biases, the compliance rate difference between μEMA and EMA was not statistically significant for *completed* participants. Even if survivor bias existed, they sustained higher μEMA interruption rates without external rewards, achieving response rates comparable to EMA. We attribute the high response rate of μEMA to its cognitive simplicity enabled by microinteraction-style question-answering on the smartwatch that enables the “interrupt more, ask less” way of collecting self-report data. The μEMA performance suggests that it might be viable for large-scale intensive longitudinal data collection studies where monetarily compensating participants in the long term may not be practical. In fact, throughout the study duration, μEMA completion rate remained consistently higher than EMA for the *completed* participants (Figure 6, Appendix A).

The difference between compliance and completion rates is greater for μEMA than for EMA (Figure 3). Compliance includes all scheduled prompts, and undelivered prompts often occur due to devices being off, in energy saver mode, or “do not disturb” mode. Smartwatches, as smartphone extensions, aren’t designed for prolonged interactions [41]. Notifications in low-battery or energy-saving mode are less noticeable on smartwatches than smartphones. Additionally, smartphone charging is more habitual at home, work, or when stationary, while smartwatches require dedicated hardware that limits charging options. Nevertheless, the 12% gap between μEMA’s compliance and completion rate among the *completed* participants shows that μEMA prompts once delivered are likely more noticeable (because of vibrotactile feedback on the wrist) than phone prompts and may also be easier to answer with a quick microinteraction.

**Fig. 7.**
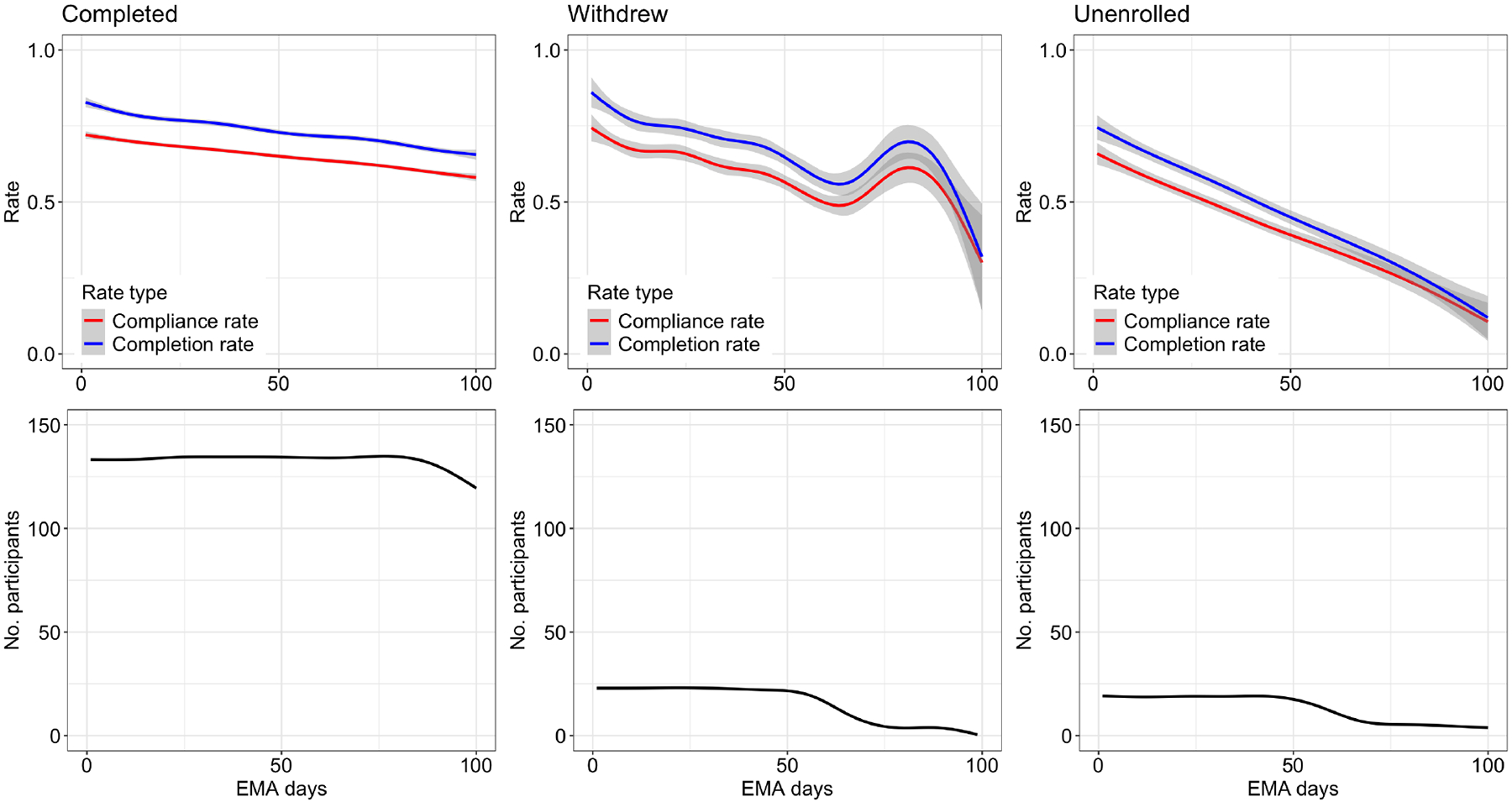
Daily trends in EMA compliance and completion rates for *completed*, *withdrew*, and *unenrolled* participants. EMA data collection takes place over 100 days (across different bursts), making the 50th day the sixth month of data collection in TIME study.

Compared to *completed* participants, μEMA outperformed EMA among *withdrew* and *unenrolled* participants. Statistically significant but marginal, *withdrew* participants were only 1.04 times as likely to answer μEMA prompts than EMA prompts. Participants voluntarily withdrew from the study, citing reasons that included changes in devices, travel, and changes in lifestyle, among others. Thus, it is possible that maintaining both the smartphone and smartwatch to regularly receive scheduled prompts was challenging, impacting the compliance rate. Yet, those who *withdrew* were 1.67 times as likely to answer delivered μEMA prompts vs. EMA prompts. Further, the μEMA compliance and completion rates for *withdrew* participants dropped much later in the study (Figures 6 and 7; Appendix) compared to EMA. Despite the challenges of continuing data collection before deciding to voluntarily withdraw, *withdrew* participants could still sustain μEMA for longer after six months.

The largest gap between μEMA and EMA response rates occurred among unenrolled participants — those unenrolled by staff for poor EMA response rates. Despite no eligibility impact from μEMA performance or active nudging, unenrolled participants were 2.25× more likely to respond to delivered μEMA prompts than EMA. The always-accessible nature of smartwatches likely makes μEMA questions easier to access and answer when prompted. EMA surveys may be missed when users are either away from their phones or put them in inaccessible locations. The daily response rate trends for *unenrolled* participants (See Figures 5 and 6 in Appendix) also suggest that EMA response rates declined more steadily than μEMA for both *withdrew* and *unenrolled* participants. These results highlight that engagement with μEMA can be sustained longitudinally, especially among participants who could sustain EMA beyond six months of data collection.

### 5.2 μEMA Less Burdensome Regardless of Engagement with EMA

In the TIME study, perceived burden was measured at six months and study-end (12 months). The six-month assessment tested whether μEMA/EMA burden predicted continued participation in the remaining six months. Our model showed two main effects (no significant EMA type × participant status interaction): First, μEMA was perceived as less burdensome than EMA, consistent with prior studies, likely due to its microinteraction design requiring only glanceable responses; yes/no questions in μEMA reduced cognitive burden despite its more interruptive schedule.

Second, *completed* participants reported lower burden (regardless of EMA type) at six months than those who later *withdrew* or were *unenrolled*. *Withdrew* participants perceived both μEMA and EMA as more burdensome than *unenrolled* participants. The withdrawal probably resulted from the burden of EMA or lifestyle changes that hindered participation, while *unenrolled* participants were removed by staff. Burden evaluation may have influenced *withdrew* participants’ voluntary exit. Thus, response rates and perceived burden together holistically clarify engagement levels of *withdrew* and *unenrolled* participants with μEMA in intensive longitudinal data collection. Finally, among the *completed* participants, μEMA was perceived to be less burdensome than EMA despite higher daily interruptions, more days of exposure, no external motivators (e.g., monetary compensation), and comparable compliance rates. These results suggest μEMA enables temporally dense self-reports in intensive longitudinal studies while maintaining favorable response rates and manageable burden.

### 5.3 Research and Design Implications of μEMA

Our findings have several implications for research in HCI, mobile sensing, and health behavior research.

#### 5.3.1 Personalized and High-resolution Data Collection.

μEMA may enable more personalized data collection in longitudinal studies by supplementing EMA, especially if/when users start disengaging with EMA. Users may also be more receptive to μEMA questions than EMA surveys in certain contexts (e.g., in transit), where obtaining EMA engagement is difficult [66]. In future intensive longitudinal studies, real-time machine learning approaches might be used to identify optimal moments to prompt self-reports from both μEMA and EMA (balancing for burden and information depth). μEMA and EMA might be combined strategically: detailed information might be first collected in bursts via EMA (as in the TIME study), and responses from EMA surveys might then trigger appropriate single-item μEMA questions to validate behavior models or capture high-frequency data on key constructs (e.g., [53]). Additionally, μEMA can be explored as a lightweight, low-burden data collection for populations less able to engage regularly with EMA (e.g., unenrolled participants in the TIME study), such as night shift workers or those with highly mobile jobs where phones are not always accessible. Moreover, beyond smartwatches, μEMA might be deployed on eyewear or headsets for more multimodal input (e.g., [52]).

#### 5.3.2 Just-in-Time Adaptive Interventions.

With a finer-grained prompting schedule, μEMA can both collect real-time behavioral data for just-in-time interventions and evaluate their effectiveness in the real world. For example, μEMA might be used to capture momentary mood (e.g., stress), recommend context-appropriate exercises, and then follow up with a quick question about how the user feels. Repeating these microinteraction loops could help optimize interventions over time. This approach could be especially valuable in microrandomized trials, where individuals are randomized many times with different interventions [45]. In such cases, sustained engagement made possible via μEMA’s low burden could enable continuous, personalized evaluation and optimization. Combined with passively collected context data (e.g., movement, location, time of day), μEMA could be triggered at opportune moments to reduce burden and maximize information gain to tailor interventions.

#### 5.3.3 Real-time Human Feedback for Novel Interfaces.

Beyond health and behavioral science, μEMA could be used to collect human feedback in real time to personalize user interfaces and experiences. For example, μEMA might capture subjective variables like user satisfaction at key points in the user journey, enabling continuous usability assessment. Beyond usability, μEMA might be used to gather feedback on content quality, perceived relevance, and trust in recommender systems (where self-report evaluations are gaining traction) [99]. Moreover, many foundational models are fine-tuned using human feedback, but traditional thumbs-up/down ratings provide limited insight. μEMA’s low-burden, context-rich questioning might be adapted to capture more detailed feedback, longitudinally, to better personalize models [12].

## 6 LIMITATIONS

This work has several limitations that create opportunities for future research. First, because the study is focused on young adults (18–29 years old) who may be used to interruptions from their mobile devices, our conclusions may not be generalizable to other populations. We compared μEMA only with traditional phone-based EMA, where the question set was randomly presented after a prompt. It may be worthwhile to compare μEMA with other low-burden EMAs such as the lockscreen-based EMA [84] and the gamified EMA [6, 46]. Both designs offset the user burden by reducing the response burden (with a single question on the lock screen) or by using novel game mechanics.

The TIME study is biased toward participants who completed the initial EMA burst and received a smartwatch for μEMA. We also limited our analysis to those who provided at least six months of data. While compliance monitoring by TIME study staff led many participants to withdraw or be unenrolled [92], this selection bias enabled testing longitudinal μEMA engagement against EMA for participants with a reasonable baseline EMA compliance. In fact, most EMA studies last only seven days [95], where novelty may drive engagement. However, our six-month (and longer) duration reduces novelty effects for both μEMA and EMA. Most of the data collection from the TIME study took place during 2020–2021, when COVID-19 restrictions were in place, limiting the mobility of the participants.

The study data collection was mainly focused on affect-based variables. There is an opportunity to replicate this work in other domains where μEMA has been used for longitudinal data collection, such as audio experiences [96] and suicidal ideation [47], among others. When comparing μEMA and EMA responses, we relied solely on within-participant variance across constructs. However, this method may be biased due to missing data or skewed responses. Exploring alternatives such as intraclass correlations or location-scale modeling [21] could enable more granular temporal comparisons. Opportunities also exist to explore temporal associations between μEMA and EMA response rates (e.g., using Granger Causality [58]) to determine if engagement shifts in one influence the other. Moreover, this study only explored the association between data collection method (μEMA or EMA) and user engagement (response rates and perceived burden). Future research can explore more machine learning based approaches to predict and optimize user engagement based on prior response rates and burden. Similar efforts have been made in prior research to not only adopt EMA prompt timing [40], but also to select questions personalized to context using real-time pattern recognition algorithms [53]. Finally, future research could include in-depth qualitative studies of engagement with μEMA and EMA in intensive longitudinal data collection.

## 7 CONCLUSION

Microinteraction-EMA enables high-density in-situ self-reports via single questions with yes/no type answers that can be completed with a quick glanceable microinteraction on a smartwatch. We evaluated μEMA’s longitudinal user engagement using data from a large-scale intensive longitudinal data collection study. We compared μEMA and EMA response rates and perceived burden across three participant categories (defined by their engagement with EMA at the end of the study) — *completed*, *withdrew*, and *unenrolled* participants. Despite μEMA’s higher interruptions, the completion rate difference between μEMA and EMA was greater for *withdrew* and *unenrolled* participants than *completed* participants. On the other hand, regardless of the participant status at the end of the study, μEMA was perceived as less burdensome than EMA. Moreover, *withdrew* participants reported a higher perceived burden than *completed* or *unenrolled* participants. These results suggest μEMA could serve as an EMA alternative for participants struggling with sustained EMA use in longitudinal studies, and as a low-burden complement to EMA, enabling dense μEMA data collection between shorter EMA bursts.

## Figures and Tables

**Table 1.** Summary of μEMA and EMA data collection in the TIME study.

	EMA (Smartphone)	μEMA (Smartwatch)
Prompts/hour	1	4
Questions/prompt	18+	1
Example answers/question	“Extremely, Quite a bit, Moderately, A little, Not at all”	“Yes, Sort of, No”
Prompts/day (assuming 8hrs. of sleep)	15	62
Days/month	8	22 or 23

**Table 2.** Demographics in the TIME study for those who completed at least six months of data collection.

Demographics	All (N=176)	*Completed* (N=134)	*Withdrew* (N=23)	*Unenrolled* (N=19)^+^
Age in Years (Mean ± SD)	23.4 (3.2)	23.5 (3.2)	23.3 (3.3)	22.7 (3.2)
Sex (n (%))				
Male	76 (43.4)	57 (42.5)	12 (52.2)	7 (38.9)
Female	99 (56.6)	77 (57.5)	11 (47.8)	11 (61.1)

+ One unenrolled participant did not complete the demographic survey.

**Table 3.** Compliance and completion rate summaries across *completed*, *withdrew*, and *unenrolled* participants.

	Completed (N=134)		Withdrawn (N=23)		Unenrolled (N=19)	
	EMA	μEMA	EMA	μEMA	EMA	μEMA
Overall compliance rates %	65.42	63.99	62.06	63.12	46.50	56.01
Overall completion rates %	72.47	80.81	69.93	79.54	52.21	70.22
Mean participant compliance rate % (SD)	65.93 (13.27)	64.02 (19.86)	62.32 (11.41)	63.01 (14.19)	48.31 (14.60)	57.64 (14.22)
Mean participant completion rate % (SD)	72.71 (14.62)	79.78 (13.85)	69.88 (12.68)	78.29 (12.14)	53.89 (17.12)	70.59 (14.56)
Mean day-level compliance rate % (SD)	64.82 (20.64)	64.41 (27.81)	61.43 (21.90)	63.44 (24.98)	45.31 (25.62)	55.92 (27.00)
Mean day-level completion rate % (SD)	73.09 (22.88)	74.62 (26.58)	70.12 (24.42)	77.16 (21.98)	51.81 (29.19)	66.67 (26.51)

**Table 4.** Coefficients of the compliance and completion rate models obtained from logistic regression. The participant status is dummy-coded with the *completed* category as a reference.

	Compliance model			Completion model		
Variable	Estimate	Std. Error	Z value	Estimate	Std. Error	Z value
Intercept (*β*_0_)	0.70*	0.07	9.74	1.13*	0.07	15.67
Method (EMA as reference)						
μEMA (*β*_1_)	−0.11*	0.01	−20.75	0.42*	0.01	67.90
Participant status *(Completed* category as reference)						
Unenrolled (*β*_2_)	−0.81*	0.20	−3.95	−0.95*	0.21	−4.66
Withdrew (*β*_3_)	−0.17	0.19	−0.90	−0.19	0.19	−1.01
Interaction terms (μEMA * [Category])						
Unenrolled (*β*_4_)	0.52*	0.02	29.90	0.38*	0.02	19.88
Withdrew (*β*_5_)	0.15*	0.02	9.30	0.09*	0.02	4.68

* Statistically significant at p <0.001

**Table 5.** Odds ratios of completing scheduled (i.e., from compliance model) and delivered (i.e., from completion model) prompts for μEMA vs. EMA on a given day across *completed*, *withdrew*, and *unenrolled* participants (statistically significant at p<0.001).

	*Completed* ($e^{\beta_{1}}$)	*Withdrew* ($e^{\beta_{1}}*e^{\beta_{4}}$)	*Unenrolled* ($e^{\beta_{1}}*e^{\beta_{5}}$)
	OR (95% CI)	OR (95% CI)	OR (95% CI)
Compliance model	0.89 (0.88, 0.90)	1.04 (0.99, 1.08)	1.50 (1.43, 1.58)
Completion model	1.53 (1.51, 1.54)	1.67 (1.51, 1.74)	2.25 (2.13, 2.34)

**Table 6.** Perceived burden of μEMA and EMA measured after 6 and 12 months in the study via the user burden scale. The scale ranges from 19 (least burdensome) to 95 (most burdensome). Only completed participants answered the user burden scale after 12 months.

Perceived user burden(Mean (SD)	Completed		Withdrew		Unenrolled	
	EMA	μEMA	EMA	μEMA	EMA	μEMA
After 6 months*	43.96 (10.65)	39.31 (11.39)	52.05 (12.96)	44.79 (13.17)	48.75 (14.73)	40.83 (13.32)
After 12 months^+^	47.32 (12.38)	41.74 (11.18)	NA	NA	NA	NA

* statistically significant at p <0.001 with no interaction between participant status and EMA type.

+ statistically significant at p <0.001

**Table 7.** Pairwise comparison of perceived burden by participant status and EMA method type

Participant status	Pairwise difference in perceived burden (95% CI)
*Unenrolled* vs. *Completed*	3.16 (−2.66, 8.98)
*Withdrew* vs. *Completed*	6.79 (2.05, 11.53) *
*Withdrew* vs. *Unenrolled*	3.63 (−3.46, 10.72)
EMA method	Pairwise difference in perceived burden (95% CI)
μEMA vs. EMA	−5.24 (−7.84, −2.63) *

* Difference statistically significant at adjusted p <0.001

## CCS Concepts:

- Human-centered computing → Empirical studies in ubiquitous and mobile computing.

## Footnotes

1
https://timestudydocumentation.github.io/docs/build/html/index.html

## A Appendix

**Table 8.** Interruption burden items added to the user burden scale [79] based on the prior work [33]. Here X is replaced with smartwatch (for μEMA) and smartphone (for EMA)

Surveys on [X] interrupted my daily activities	Not at all	A little bit	Somewhat	Very much so	Extremely
Surveys on [X] distracted me from my daily activities	Not at all	A little bit	Somewhat	Very much so	Extremely

**Table 9.** Summary of variances across EMA and uEMA and Pearson correlations across the common constructs between uEMA and EMA within-participant variances. All correlations are statistically significant at (p <0.001)

	Mean of within-participant variance (SD)			Pearson r	
Construct	μEMA	3-point EMA	5-point EMA	3-point EMA	5-point EMA
Sadness	0.30 (0.19)	0.17 (0.12)	0.44 (0.45)	0.64	0.64
Happiness	0.36 (0.15)	0.21 (0.10)	0.61 (0.36)	0.47	0.46
Fatigue	0.41 (0.17)	0.26 (0.13)	0.76 (0.43)	0.65	0.62
Relaxation	0.39 (0.18)	0.22 (0.12)	0.72 (0.44)	0.53	0.52
Tension	0.35 (0.18)	0.23 (0.13)	0.60 (0.46)	0.58	0.54
Stress	0.37 (0.16)	0.23 (0.12)	0.60 (0.49)	0.58	0.55
Frustration	0.36 (0.17)	0.24 (0.14)	0.62 (0.50)	0.58	0.57
Nervousness	0.31 (0.19)	0.21 (0.13)	0.53 (0.63)	0.58	0.56
Focus	0.35 (0.17)	0.22 (0.12)	0.75 (0.51)	0.52	0.52
Control	0.30 (0.17)	0.19 (0.12)	0.68 (0.51)	0.55	0.52
Routine	0.42 (0.18)	0.30 (0.16)	1.00 (0.64)	0.67	0.65

**Table 10.** Remaining socio-economic variables in the TIME study for those who completed at least six months of data collection.

Demographics	All (N=176)	*Completed* (N=134)	*Withdrew* (N=23)	*Unenrolled* (N=19)^+^
Ethnicity (n (%))				
Hispanic	53 (30.3)	43 (32.1)	7 (30.4)	3 (16.7)
Non-Hispanic	122 (69.7)	91 (67.9)	16 (69.6)	15 (83.3)
Race (n (%)) *				
White	85 (48.3)	62 (46.3)	15 (65.2)	8 (42.1)
Asian	71(40.3)	58 (43.3)	7 (30.4)	7 (36.8)
Black	25 (14.2)	18 (13.4)	3 (13.0)	4 (21.1)
American Indian/Alaska Native	6 (3.4)	6 (4.5)	0 (0)	0 (0)
Pacific Islander	4 (2.3)	3 (2.24)	0 (0)	1 (5.3)
Education (n (%))				
High School	18 (10.3)	12 (9.0)	3 (13.0)	3 (16.7)
Some College	76 (43.4)	57 (42.5)	9 (39.1)	10 (55.6)
College Graduate	81 (46.3)	65 (48.5)	11 (47.8)	5 (27.8)
Work Status (n (%))				
Employed	90 (51.1)	71 (53.0)	10 (43.5)	9 (47.4)
Self-employed	9 (5.1)	6 (4.5)	1 (4.4)	2 (10.5)
Out of Work	24 (13.7)	17 (12.7)	3 (13.0)	4 (21.1)
Student	101 (57.4)	77 (57.5)	12 (52.2)	12 (63.2)
Homemaker	3 (1.7)	1 (0.8)	1 (4.4)	1 (5.26)
Unable to Work	6 (3.4)	4 (3.0)	1 (4.4)	1 (5.3)
Marital Status				
Never Married	132 (75.0)	100 (74.6)	17 (73.9)	15 (79.0)
Unmarried Couple	26 (14. 8)	19 (14.2)	3 (13.0)	4 (21.1)
Married	21 (11.9)	18 (13.4)	3 (13.0)	0 (0)
Divorced	1 (0.6)	1 (0.8)	0 (0)	0 (0)

* **Note:** Participants could choose from multiple options on race and ethnicity,

+ One *unenrolled* participant did not complete the demographic survey.
